# Perfluorinated Organosilicons Enabling Low‐Loss Ferroelectric Polymer Composites for Efficient Energy Storage and Electroluminescence

**DOI:** 10.1002/advs.202414380

**Published:** 2025-03-06

**Authors:** Li Li, Zhubing Han, Hemant P Yennawar, Yunyun Cheng, Ting Han, Rui Feng, Yang Zhang, Guanghui Zhao, Qing Wang, Lijie Dong

**Affiliations:** ^1^ Center for Smart Materials and Devices State Key Laboratory of Advanced Technology for Materials Synthesis and Processing Wuhan University of Technology Wuhan Hubei 430070 P. R. China; ^2^ Department of Materials Science and Engineering The Pennsylvania State University University Park PA 16802 USA; ^3^ Department of Biochemistry and Molecular Biology The Pennsylvania State University University Park PA 16802 USA; ^4^ Research Center for Materials Genome Engineering International Materials Science and Engineering Wuhan University of Technology Wuhan Hubei 430070 P. R. China

**Keywords:** electroluminescence, energy storage, ferroelectric polymer, organosilicon

## Abstract

Ferroelectric polymers for energy storage and conversions suffer from high energy losses. Despite great efforts in polymer composites with organic or inorganic fillers, limited successes are achieved with an often compromised dielectric constant (*K*). Here, a synthesized organic–inorganic hybrid—perfluorinated polyhedral oligomeric silsesquioxane (F‐POSS) is presented for creating an ultralow‐loss ferroelectric polymer composite. The incorporation of such perfluorinated organosilicons with unique “cage‐arm” structure into the polymer not only modulates chain conformations but also inhibits charge transport through its wide bandgap and strong carrier‐trapping capabilities. This results in a significant reduction of dielectric/conduction losses and enhanced electric breakdown strength while maintaining a high *K*, yielding a discharged energy density (*U*_e_) of 22.3 J cm^−3^ with a high efficiency (*η*) of 82.3%. The utility of F‐POSS is further demonstrated in sandwiched devices (with high‐*K* layers comprising quantum dots) that deliver a high *U*_e_ of over 32 J cm^−3^ and a *η* > 80%, alongside an efficient alternating current–driven electroluminescence (luminance > 670 cd m^−2^ at 10 MV m^−1^). This work presents a facile strategy for achieving high‐*K*, low‐loss ferroelectric polymers, broadening their applications toward advanced energy storage and optoelectronic technologies.

## Graphical abstract

Ferroelectric polymers for energy storage and conversion are suffering from severe energy losses despite their highly electroactive functions. Here an organic–inorganic hybrid—perfluorinated polyhedral oligomeric silsesquioxane (F‐POSS) is synthesized to markedly suppress losses in ferroelectric polymers for achieving efficient energy storage and electroluminescent applications.

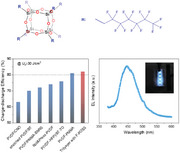

## 1 Introduction

Ferroelectric polymers, known for their spontaneous polarization, flexibility, and facile processability, are highly desirable in various energy storage and conversion fields, including electrostatic film capacitors, electromechanical sensors, alternating current‐driven electroluminescent devices (ACEL), etc.^[^ ^1^, ^2^, ^3^, ^4^ ^]^ For instance, the typical ferroelectric polymers, poly(vinylidene fluoride)(PVDF) and its copolymers like poly(vinylidene fluoride‐*co*‐hexafluoropropylene)[P(VDF‐HFP)], have shown promise in dielectrics for capacitive energy storage due to their relatively high dielectric constant (*K*, 7–14) and the positive correlation between *K* and energy density (*U* = ∫*D*d*E*, where D = K^*^E is electric displacement, *E* is applied electric field), compared to other dielectric polymers such as polypropylene (PP), polyimide (PI) (*K*< 4).^[^ ^2^, ^5^, ^6^, ^7^ ^]^ The high *K* of ferroelectric polymers also allows for the redistribution of local electric fields, promoting efficient photovoltaics and electroluminescence.^[^ ^4^, ^8^, ^9^ ^]^ However, the *K* of ferroelectric polymers falls far behind their ceramic ferroelectric counterparts, e.g., BaTiO_3_, Pb(Zr, Ti)O_3_ with a *K* exceeding 100.^[^ ^7^, ^10^ ^]^ More critically, their intrinsically high energy losses (such as hysteresis loss and conduction loss), compared to linear dielectric polymers, usually lead to early electric breakdown failure and low charge–discharge efficiency (*η*), posing significant threats to the performance and lifespan of the devices.

Efforts to tackle these issues have been long focused on the formation of ferroelectric polymer‐based composites with inorganic or organic fillers due to considerations of combining individuals’ advantages and tuning microstructures in the composites. Among them, incorporating high‐*K* ceramic fillers, e.g., BaTiO_3_, and TiO_2_, into the ferroelectric polymer was the most commonly used strategy for obtaining high‐*K* composites.^[^ ^11^, ^12^, ^13^, ^14^, ^15^ ^]^ Unfortunately, the enhancement of *K* is generally achieved at the cost of deteriorated electric breakdown strength (*E*_b_) and *η* due to large dielectric mismatches between organic polymers and inorganic fillers and defects at polymer/filler interfaces.^[^ ^16^, ^17^, ^18^, ^19^, ^20^ ^]^ Moreover, a high filling ratio of inorganic fillers (>10 vol%) is usually required for achieving optimal increase of *K*, which further damages *E*_b_ and impairs the inherent mechanical and processing advantages of polymers.^[^ ^20^, ^21^ ^]^ While recent work utilizing wide‐bandgap inorganic fillers such as BN, and Al_2_O_3_ nanoplates can improve electrical resistivity and *E*_b_,^[^ ^22^, ^23^, ^24^ ^]^ this method often compromises dielectric constant and brings scalability issues. As an alternative, ferroelectric polymer composites (or blends) with organic inclusions offer a reliable solution due to their high insulating properties and good inter‐phase compatibility, showing reduced conduction loss and improve *η*.^[^ ^25^, ^26^, ^27^, ^28^, ^29^ ^]^ However, these all‐organic composites usually exhibit a traded *K* due to much lower *K* (generally < 4) and high ratios (e.g., >40 wt.%) of the incorporated organic phases. More recently, the introduction of ultralow contents (<1 vol%) of some inorganic nanofillers like Cd_1‐x_Zn_x_Se_1‐y_S_y_, TiO_2_ in ferroelectric polymers encouragingly achieve concurrent enhancements in *K* and *E*_b_, owing to the local structural changes including stabilized polar phases and improved dipole activity at polymer‐filler interfaces.^[^ ^30^, ^31^ ^]^ Nonetheless, these composites still suffer from low *η*, especially at high electric fields (e.g., *η*≈70% at 500 MV m^−1^), which originate from the inherently high energy loss of ferroelectric polymers and organic–inorganic incompatibility. This means that a large ratio of charged energy is dissipated in the form of heat, adding risks to device performance and reliability. Hence, despite the great advancements achieved, it is still urgent and fundamentally challenging for achieving low loss and high *E*_b_ in ferroelectric polymers while maintaining their high *K*, for the realization of efficient and reliable energy storage and conversion.

In this work, we depart from conventional filler strategies by synthesizing an organic–inorganic hybrid material—perfluorinated polyhedral oligomeric silsesquioxane (F‐POSS). Unlike other organosilicon compounds that are generally amino‐functionalized and suitable for low‐*K* applications,^[^ ^32^, ^33^ ^]^ the incorporation of F‐POSS into P(VDF‐HFP) creates a new class of ferroelectric polymer composites that achieve significant improvements in *E*_b_ and *η* while enhancing *K*. The rationale behind this approach lies in the unique design of a perfluorinated “cage‐arm” framework, which not only promotes favorable interactions with the ferroelectric polymer, thereby modulating chain conformations and phase transitions but also offers a wide bandgap and strong carrier‐trapping capabilities, effectively inhibiting charge transport. This combination markedly reduces conduction loss and enhances electric breakdown strength, resulting in superior capacitive performance including ultrahigh energy density and efficiency at high electric fields, as well as efficient electroluminescence in a tri‐layer structured ACEL device. This work reveals for the first time the effectiveness of perfluorinated organosilicons—in tuning ferroelectric properties for designing high‐*K*, low‐loss ferroelectric polymers, demonstrating its significant potential for advanced energy storage and conversion applications.

## 2 Results and Discussion

### 2.1 Synthesis and Characterization of F‐POSS

**Figure 1:**
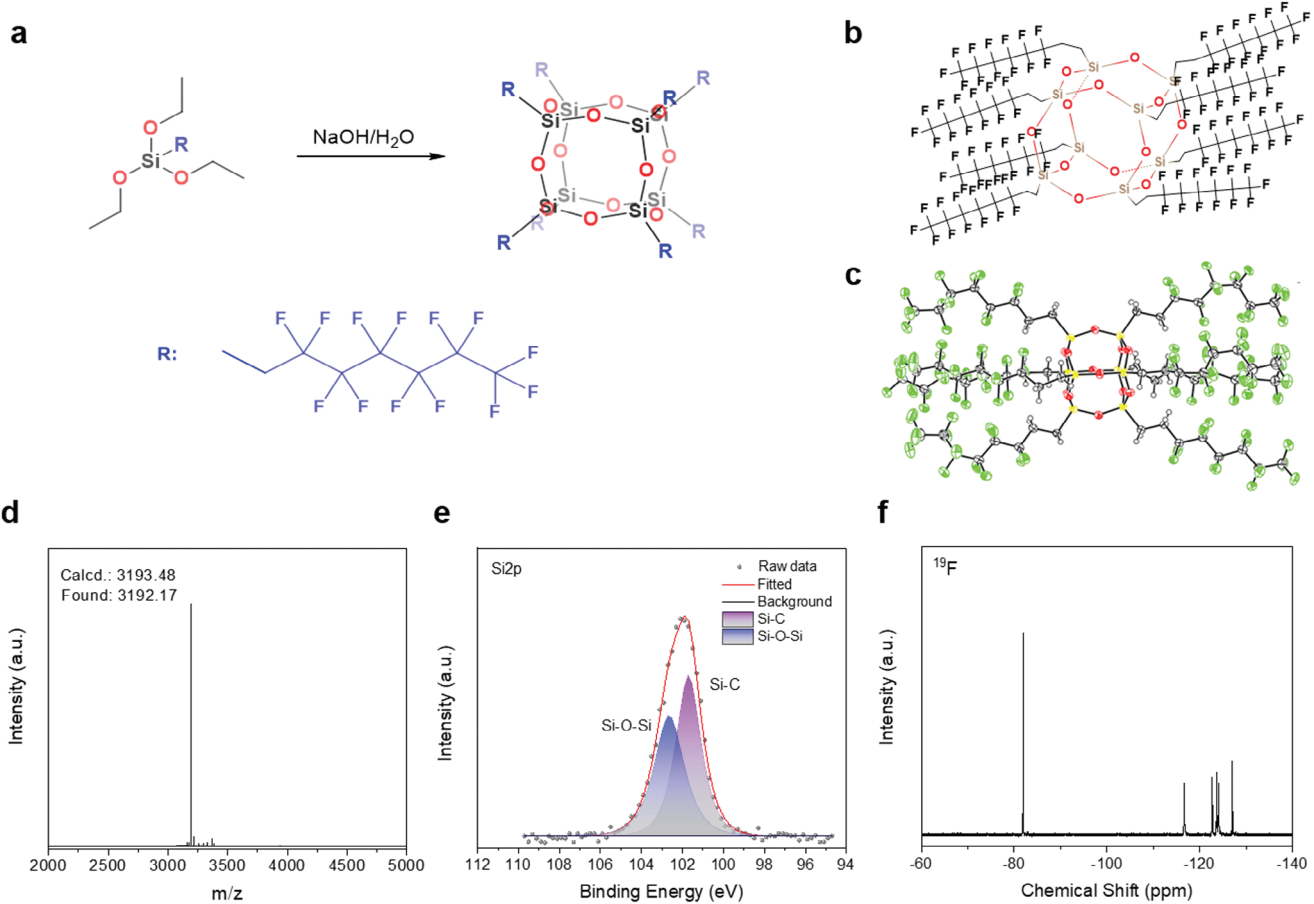
a) Synthesis route of F‐POSS. b) Schematic molecular structure of F‐POSS. c) Atomic position‐refined molecular structure of F‐POSS determined from single‐crystal XRD data. d) MALDI‐TOF mass spectrum, e) high‐resolution XPS spectrum of Si2p, and f) ^19^F NMR spectrum of F‐POSS.

F‐POSS was synthesized via a base‐catalyzed condensation of fluoroalkyl trialkoxysilanes, as depicted in **Figure** **1**a. The facile hydroxylation of trialkoxysilanes, coupled with the large size and strong electronegativity of the fluoroalkyl groups, results in a “cage‐arm‐like” organic–inorganic hybrid structure, with the core cage consisting of a Si─O─Si network, while silicon atoms armed to the eight fluoroalkyl groups (Figure 1b).^[^ ^34^ ^]^ Specifically, the spatial hindrance created by the bulky fluoroalkyl groups attached to the silicon atoms limits the possibility of extended network formation—promoting the formation of discrete, well‐defined polyhedral cages rather than continuous silica networks like polysilane. Moreover, the highly electronegative fluorine atoms in the fluoroalkyl chains create strong electron‐withdrawing effects. This would enhance the partial positive charges on the silicon atoms, facilitating strong Si─O─Si bond formation during the condensation of trialkoxysilanes, which further ensures that the resulting structure is stabilized as a closed cage.^[^ ^34^ ^]^ The element compositions of the obtained fresh powders include C, H, O, and Si, as probed by scanning electron microscopy (SEM) coupled with energy dispersive spectroscopy (EDS) (Figure S1, Table S1, Supporting Information). The molecular structure of F‐POSS was then precisely determined and confirmed by single‐crystal X‐ray diffraction (SC‐XRD, Figure S2 and Table S2, Supporting Information). The calculated molecular weight (Mw, 3193.48 g mol^−1^) of this structure closely matches the measured data (Mw≈3192.17 g mol^−1^) from a matrix‐assisted laser desorption/ionization time‐of‐flight (MALDI‐TOF) mass spectrometry test (Figure 1d). The chemical structure of F‐POSS was further examined by Fourier‐transform infrared spectroscopy (FTIR) (Figure S3, Supporting Information) and X‐ray photoelectron spectroscopy (Figure 1e; Figure S3, Supporting Information), evidencing characteristic groups of F‐POSS such as ─CF_2_, ─CF_3_, and Si─O─Si. Nuclear magnetic resonance spectroscopy (NMR, ^19^F) identifies six distinct chemical shifts (Figure 1f), corresponding to the F atoms in F‐POSS, similar to previous reports on fluorinated POSS for hydrophobic studies.^[^ ^34^, ^35^ ^]^ These results collectively confirm the successful synthesis of F‐POSS. In addition, thermal characterizations (thermogravimetric analysis, TGA, and differential scanning calorimetry, DSC) reveal a melting point of 127.8 °C and a subliming temperature of ≈267.4 °C (see more discussions in Figure S4, Supporting Information), which are in alignment with other fluorinated POSS compounds.^[^ ^34^ ^]^

### 2.2 Electronic Properties of F‐POSS and Interactions with Ferroelectric Polymer

**Figure 2:**
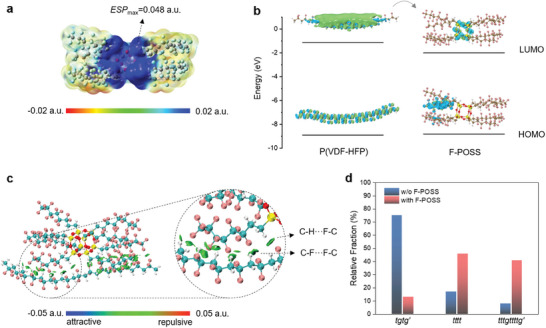
a) Electrostatic potential mapping of F‐POSS. b) Energy diagram of P(VDF‐HFP) and F‐POSS with HOMO and LUMO mappings. c) Visualization of intermolecular interactions between P(VDF‐HFP) and F‐POSS. d) Relative fractions of *tgtg’*, *tttt*, and *tttgtttg’* in P(VDF‐HFP) and the composites with 12 wt.% F‐POSS.

We next investigated the electronic properties of F‐POSS based on density functional theory (DFT) calculations. The electrostatic potential (ESP) mapping (**Figure** **2**a) indicates that F‐POSS possesses predominantly positive potentials with high ESP values on the surface of the Si─O─Si cage, signifying a strong electron‐trapping capability. DFT calculations (Figure 2b) reveal that F‐POSS has a high bandgap (≈6.8 eV), surpassing most conventional inorganic or organic fillers, such as BN (≈6.4 eV), TiO_2_ (≈3.2 eV), and PCBM (≈1.8 eV).^[^ ^36^, ^37^ ^]^ This wide bandgap is conducive to achieve inherently low dielectric loss (tan*δ* = 0.005 at 1 kHz) and high resistivity, as evidenced in Figure S5 (Supporting Information). Moreover, F‐POSS exhibits a much lower lowest unoccupied molecular orbital (LUMO) level (−2.0 eV) compared to P(VDF‐HFP) (−1.1 eV), suggestive of a high electron affinity, and thus a high electronic trap depth (≈0.9 eV) between them, while their highest occupied molecular orbital (HOMO) levels remain nearly the same. The LUMO of F‐POSS is primarily localized on the Si─O─Si cage, revealing the trapping characteristics of the inorganic framework, consistent with the ESP results. These findings manifest that the incorporation of F‐POSS would impede carrier transport and thus enhance resistance to electrical failure in ferroelectric polymer.

We then prepared P(VDF‐HFP)/F‐POSS composite films and examined their chemical structures through theoretical simulations and structural characterizations. The interactions between the polymer and F‐POSS were first explored using an independent gradient model based on Hirshfeld partition (IGMH),^[^ ^38^, ^39^ ^]^ as shown in Figure 2c. Multiple green‐colored ellipsoids, which represent weak attractive intermolecular interactions, including C─F···H─C bonding and C─F···F─C halogen bonding, are identified between F‐POSS and P(VDF‐HFP) (using an all*‐trans* chain as an example). Here the weak halogen bonding likely arises from a combination of electrostatic and dispersion forces,^[^ ^40^ ^]^ which are consistent with studies on halogen bonding in fluorinated systems, where C─F···C─F contacts contribute to molecular packing and interfacial stability.^[^ ^34^, ^41^ ^]^ These interactions suggest good compatibility between F‐POSS and P(VDF‐HFP), which is supported by the optical and SEM images of the composite films (Figures S6 and S7, Supporting Information), displaying a homogeneous morphology without aggregations or voids. More importantly, we find that these interactions potentially induce conformational transformations—from *tgtg’* to *tttt* (or all *trans*) or *tttgtttg’* in the ferroelectric polymer. Higher binding energies exist between F‐POSS and polar conformations compared to that with the non‐polar one, i.e., −19.4, −18.6, and −5.2 kcal mol^−1^ for *tttt*, *tttgtttg’*, and *tgtg’*, respectively (Figure S8, Supporting Information). This is experimentally verified by FTIR analyses, where the relative conformational ratios are determined by integrating their characteristic IR peaks (Figure S9, Supporting Information).^[^ ^30^, ^42^ ^]^ It is shown that compared with pristine P(VDF‐HFP) the fractions of *tttt* and *tttgtttg’* increase from 17% to 46% and 8% to 41%, respectively, while *tgtg’* decreases from 75% to 13%, in the composite with 12 wt.% F‐POSS (Figure 2d). Such non‐polar to polar transitions have also been verified in XRD patterns (Figure S10, Supporting Information). These improved fractions of polar phases are anticipated to facilitate a high dielectric constant and electric displacement, with the enhanced *γ* phase being more favorable for achieving low ferroelectric hysteresis loss under high electric fields.^[^ ^14^, ^30^, ^42^, ^43^ ^]^ In addition, the incorporation of F‐POSS promotes the crystallization of the polymer, with crystallinity increasing from ≈27% of pristine P(VDF‐HFP) to ≈38% of the composite with 12 wt.% F‐POSS, as derived from DSC curves (Figure S11, Supporting Information), which further enlarges the contributions of polar phases.

### 2.3 Dielectric and Energy Storage Properties

**Figure 3:**
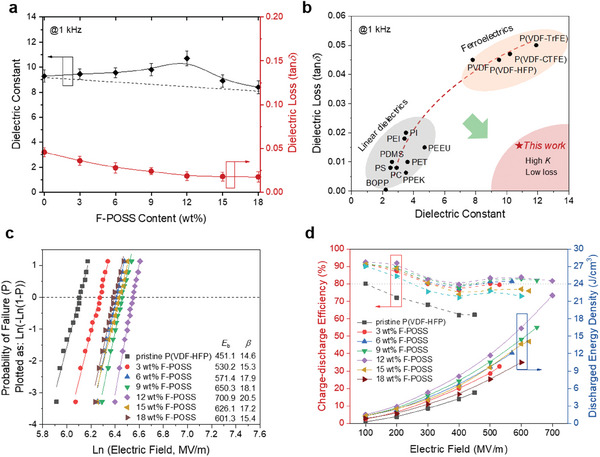
a) Dielectric constant and loss at 1 kHz of P(VDF‐HFP) composites as a function of F‐POSS content. b) Comparison of dielectric constant and loss between this work and other reported results. c) Weibull breakdown plots of pristine P(VDF‐HFP) and composite films with varied F‐POSS contents. d) Charge–discharge efficiency and discharged energy density as a function of the electric field of pristine P(VDF‐HFP) and composite films with varied F‐POSS contents.

We then investigated the dielectric properties of P(VDF‐HFP)/F‐POSS composites. As seen in **Figure** **3**a and Figure S12 (Supporting Information), *K* (at 1 kHz) of the composite films increases from P(VDF‐HFP) (≈9.1) to a maximum of ≈10.7 at 12 wt.% F‐POSS loading, despite the much lower *K* (≈3.8) of F‐POSS (Figure S5, Supporting Information). This enhanced *K* does not conform to the classic two‐phase dielectric models,^[^ ^44^, ^45^, ^46^, ^47^ ^]^ which are formulated based on volumetric summations of individual components, and has rarely been achieved in polymer composites with POSS derivatives. Here, the dielectric constant is a competitive result from the positive contributions of polar phases^[^ ^30^, ^31^ ^]^ and the negative one of low‐*K* F‐POSS. At low F‐POSS contents (below 12 wt.%), the greatly increased polar phases dominate the negative contribution from the low‐*K* F‐POSS, thereby leading to the dielectric enhancement. Further increasing F‐POSS content to 18 wt.% results in a reduction of K to 8.2 in the composite films, due to the greater volumetric contributions of the low‐K F‐POSS, while decreased crystallinity and stabilized relative polar phase fractions (Figures S9–S11, Supporting Information). As a result of the low loss and high insulating properties of F‐POSS, as well as improved dipole rotations enabled by the conformation changes, tan*δ* (at 1 kHz) of the composite films markedly decreases from ≈0.045 of pristine P(VDF‐HFP) to ≈0.016 of the composite with 12 wt.% F‐POSS (Figure 3a; Figure S12, Supporting Information). Such a combination of high *K* and low loss makes this material potentially desirable for practical use, compared to current dielectric polymers including ferroelectric polymers (high *K* yet high loss) like PVDF and linear dielectric polymers (low loss but also low *K*) such as biaxially oriented polypropylene (BOPP), polystyrene (PS) and polyetheretherketone (PEEK), etc. (Figure 3b).^[^ ^7^ ^]^ The high‐field electric leakage current (*J*) also decreases significantly in the composites, with *J* being 2.6 × 10⁻^7^ A cm^−^^2^ at 300 MV m^−1^ for the composite film with 12 wt.% F‐POSS, nearly an order of magnitude lower than that of pristine P(VDF‐HFP) (2.4 × 10⁻^6^ A cm^−2^) (Figure S13, Supporting Information). This mainly results from the strong charge‐trapping features and wide bandgap of F‐POSS that efficiently restrict charge transport. Furthermore, the introduction of F‐POSS significantly enhances the mechanical properties of the ferroelectric polymer, with Young's modulus (*Y*) increasing from 0.53 GPa of pristine P(VDF‐HFP) to 1.21 GPa of the composite with 12 wt.% F‐POSS (Figure S13, Supporting Information), primarily due to enhanced crystallinity thus dense chain‐packing induced by F‐POSS, which may act as nucleating agents for the polymer, considering the inter‐molecular interactions between them. Such contributions overweigh the potentially negative effects of the voids in the F‐POSS structure, which are generally regarded as weak points, but show limited influence due to the small sizes (≈0.027 nm^3^) and rigid Si–O–Si network.^[^ ^34^, ^41^ ^]^ The decrease in *Y* with further increasing F‐POSS content is due to the decreased crystallinity while increased ratios of intrinsic voids of F‐POSS. Both the suppressed leakage current and improved mechanical strength are beneficial for achieving high electric breakdown strength in the composites, according to classic electric conduction and electromechanical breakdown mechanisms.^[^ ^30^, ^42^ ^]^

We then examined the electric breakdown characteristics of various P(VDF‐HFP)/F‐POSS composites based on two‐parameter Weibull statistics,^[^ ^48^ ^]^ represented by the equation: $P\;\left(E\right)=1-\exp\left[{\left(-E/E_{b}\right)}^{\beta }\right]$, where *P(E)* represents the cumulative probability of electric failure, *E* is the measured electric breakdown strength, *E*_b_ is the characteristic breakdown strength corresponding to a ≈63% probability of electric failure and *β* is the shape parameter indicating the data scattering. As presented in Figure 3c, *E*_b_ increases remarkably from 451.1 MV m^−1^ of pristine P(VDF‐HFP) to 700.9 MV m^−1^ of the composite film with 12 wt.% F‐POSS. This surpasses a variety of ferroelectric polymer‐based dielectric materials (Figure S13, Supporting Information).^[^ ^1^, ^42^, ^49^, ^50^, ^51^, ^52^ ^]^ The higher *β*  values than that of pristine P(VDF‐HFP), e.g., 20.5 and 14.6 for the composite with 12 wt.% F‐POSS and pristine P(VDF‐HFP), respectively, are suggestive of enhanced reliability of the polymer composites. The improved *E*_b_ can be rationalized as follows. While the voids of F‐POSS may play a negative role in breakdown strength due to weak points, the strong charge‐trapping effects of F‐POSS arising from its large positive electrostatic potentials and the higher electron affinity lead to largely reduced high‐field conduction losses. Moreover, the increased crystallinity thus dense chain‐packing in the composites induced by F‐POSS makes a great contribution to the improved mechanical strength that is also beneficial for a high *E*_b_. Therefore, when the contents of F‐POSS are relatively low (e.g., below 12 wt.%), the combination of both positive contributions of charge trapping and enhanced mechanical strength overweigh the negative impacts of voids in F‐POSS, leading to the improved *E*_b_. Yet, with further increasing F‐POSS contents, the decreased crystallinity and enhanced leakage current coupled with increased voids lead to intensified high‐field conductions and weak electromechanical resistivity to the electrostatic forces, resulting in reduced *E*_b_.

The combination of a high *K*, high *E*_b,_ and low loss results in exceptional capacitive energy storage performance in the P(VDF‐HFP)/F‐POSS composites, as demonstrated in Figure 3d. Compared to the pristine P(VDF‐HFP), which exhibits a limited energy density (*U*_e_) of ≈5 J cm^−3^ and an efficiency (*η*) of 62% at an electric field of 450 MV/m, the composite film containing 12 wt.% F‐POSS achieves a *U*_e_ of 22.3 J cm^−3^ with a high *η* of 82.3% at a high electric field of 700 MV m^−1^. This energy storage performance is superior to those of conventional ferroelectric polymers, including PVDF, P(VDF‐HFP), and P(VDF‐TrFE‐CTFE), which are generally constrained by low breakdown strengths (<500 MV m^−1^) and/or low efficiencies (< 65%), making the P(VDF‐HFP)/F‐POSS composites standing out among the high‐*η* polymer blends or composites (Figure 3d).^[^ ^26^, ^42^, ^53^, ^54^, ^55^, ^56^, ^57^, ^58^ ^]^ For instance, P(VDF‐HFP)/PMMA blends and PVDF/glucose composites achieve an *U*_e_ of ≈11 and 21 J cm^−^^3^, respectively, at *η* ≥ 80%.^[^ ^54^, ^55^ ^]^ These results highlight the important effects of F‐POSS in overcoming high losses and improving energy storage density in ferroelectric polymer.

### 2.4 Trilayered Composites for Efficient Energy Storage and Electroluminescence

**Figure 4:**
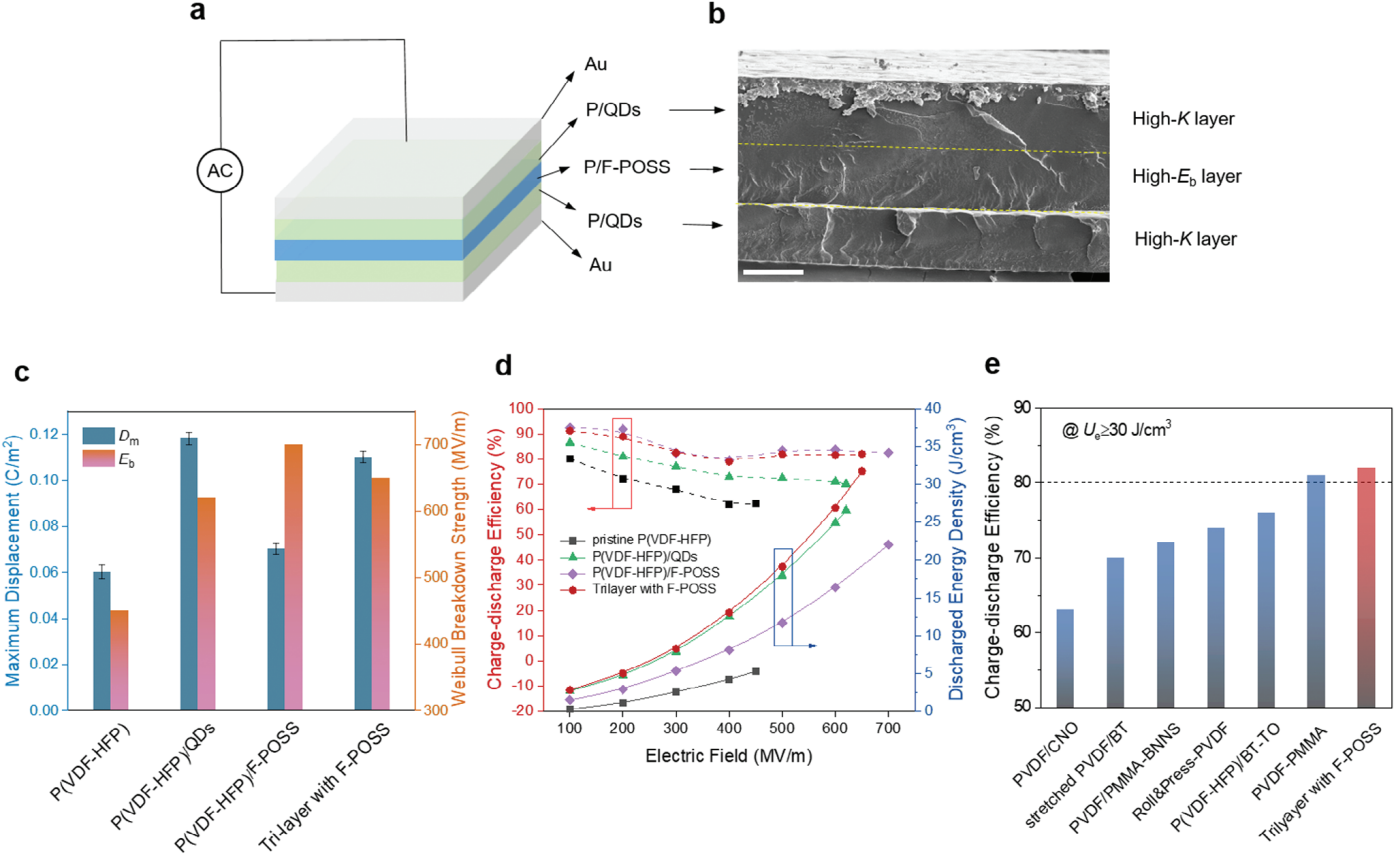
a) Schematic illustration of the trilayered composites for capacitive energy storage. P(VDF‐HFP) is simplified as P for clarification. b) Cross‐section SEM image of the sandwiched composites. Scale bar, 5 µm. c) Maximum displacement and Weibull breakdown strength and d) charge–discharge efficiency and discharged energy density of various polymers and composites. e) Comparison of charge–discharge efficiency at maximum electric fields between this work and other reported results at a criterion of energy density over 30 J cm^−3^.^[^ ^56^, ^57^, ^58^, ^60^, ^61^, ^62^, ^63^, ^64^, ^65^, ^66^ ^]^

To further demonstrate the superiority of low loss and high *E*_b_ of the new ferroelectric polymer composites enabled by F‐POSS, we prepared a tri‐layer film using a structural design that effectively redistributes electric fields across the composite (Equations S1 and S2, Supporting Information). The tri‐layer composite films for capacitors were prepared by solution‐casting individual layers combined with hot‐pressing in a designated order (see methods in Supporting information). As depicted in **Figure** **4**a, the P(VDF‐HFP)/F‐POSS middle layer is sandwiched between two identical high‐*K* layers (*K* = 18.6) composed of our previously reported P(VDF‐HFP) composites with Cd_1‐x_Zn_x_Se_1‐y_S_y_ quantum dots (QDs, Figure S14 (Supporting Information).^[^ ^30^, ^59^ ^]^ The cross‐section SEM image of the composite film (Figure 4b) reveals the tri‐layer architecture characteristics, with each layer having a controlled thickness of approximate 5–6 µm and a uniform morphology devoid of cracks or pores. Owing to the balanced *K* and *E*_b_ in this structure, both the maximum electric displacement (*D*_m_) and *E*_b_ of the composite are in line between the individual single layers, i.e., *D*_m_ and *E*_b_ are 0.094 C m^−2^ and 650 MV m^−1^ for the tri‐layer composite, compared to 0.118 C m^−2^ and 620 MV m^−1^ for the P(VDF‐HFP)/QDs layers, and 0.08 Cm^−2^ and 700 MV m^−1^ for the P(VDF‐HFP)/F‐POSS layer. Consequently, the tri‐layer composite achieves an ultrahigh *U*_e_ of ≈32.2 J cm^−3^ at a high *η* of ≈82%, which is more than 5 times higher than that of the pristine P(VDF‐HFP). Generally, achieving both high *U*_e_ and *η* is unattainable in the ferroelectric polymers and nanocomposites. For instance, the PVDF composites with Ca_2_Nb_3_O_10_ (CNO) nanosheets show a high *U*_e_ of 36.2 J cm^−3^ yet a limited *η* of 61.2%.^[^ ^60^ ^]^ The stretched PVDF/BaTiO_3_ (BT) nanocomposites exhibit a giant *U*_e_ of over 40 J cm^−3^ but accompanied by a *η* of ≈70%.^[^ ^61^ ^]^ Therefore, such a high *U*_e_ and *η* obtained in the trilayer composite places it among the best high‐efficiency ferroelectric polymer composites with high energy density reported thus far (Figure 4e).^[^ ^56^, ^57^, ^58^, ^60^, ^61^, ^62^, ^63^, ^64^, ^65^, ^66^ ^]^

**Figure 5:**
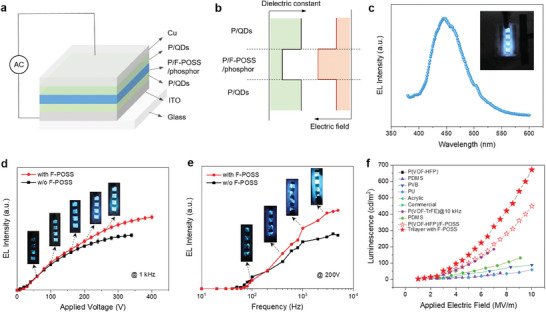
a) Schematic diagram of the sandwiched composite for electroluminescence. P(VDF‐HFP) is simplified as P for clarification. b) Schematic illustration of the distributions of dielectric constant and electric field in the sandwiched structure. c) EL spectrum of the sandwiched composite. The inset typically shows the electroluminescence of the sample. Luminance as a function of d) frequency and e) applied voltage of the sandwiched composites with and without F‐POSS. The inset pictures show the electroluminescence of the samples at certain voltages and frequencies. f) Comparison of electric field‐dependent luminance of devices established on the sandwiched composites and other polymers.^[^ ^4^, ^8^, ^9^, ^70^, ^71^, ^72^, ^73^, ^74^, ^75^, ^76^, ^77^, ^78^ ^]^

Aside from the improved energy storage, we also demonstrate the utility of F‐POSS in promoting highly efficient electroluminescence in the trilayer ferroelectric polymer composites comprising phosphor particles (ZnS:Cu, Figure S15, Supporting Information), as schemed in **Figure** **5**a. To prepare the tri‐layer composite films for electroluminescent devices (on ITO‐coated glasses), a layer‐by‐layer casting procedure was employed (see methods in Supporting information), which leads to compact and integrated composite film devices in the absence of external pressure. According to the principle of electric‐field distribution (Equations S1 and S2, Supporting Information for more discussions), the P(VDF‐HFP)/F‐POSS middle layer with a lower *K* than that of top/bottom layers is expected to experience much higher local electric fields (Figure 5b). Moreover, the electric field can be further concentrated on the phosphor particles because of their lower *K* (≈8.3) than the matrix,^[^ ^8^, ^9^, ^67^ ^]^ thereby significantly improving the EL intensity at low electric fields (or driving voltages). We prepared such tri‐layer composites with varying phosphor contents, which show good integrity and layered distribution characteristics, as evidenced in the cross‐section images and EDS mapping (Figure S16, Supporting Information). We find that the EL intensity increases with phosphor content, peaking at ≈60 wt.%, with bright blue luminescence (peak wavelength ≈450 nm) (Figure 5c). It is also evident that the device comprising F‐POSS exhibits much higher EL intensity than that without at same loadings, indicative of its high efficiency in improving luminance (Figure S17, Supporting Information). Further increases in phosphor loading result in a decline in EL intensity, possibly due to filler aggregations and increased leakage current.^[^ ^9^ ^]^ We then measured the luminance of the devices under alternating‐current conditions, finding that the luminance of the sandwiched composite with phosphors is generally proportional to the applied electric voltage (up to 300 V) and frequency (up to 5 kHz) (Figure 5d,e). The inset pictures vividly demonstrate the luminescence changes with increasing voltage and frequency. The voltage‐dependent luminance can be well fitted by the equation: $L_{v}=L_{v,0}\;\exp\left(-b/V^{0.5}\right)$ (the solid lines in Figure 5e), where *L* is the luminance, *V* is the voltage amplitude, and *L*_v_,_0_ and *b* are fitting parameters determined by the device.^[^ ^8^, ^68^ ^]^ These observations are suggestive of enhanced injection of accelerated electrons driven by alternating electric fields that activate the luminescent centers, in consistence with previous reports.^[^ ^8^, ^9^, ^69^ ^]^ Furthermore, owing to the concentrated electric fields on phosphors, the device based on the tri‐layer composite comprising F‐POSS exhibits a much lower turn‐on voltage of ≈15 V at a frequency of 1 kHz, compared to that without F‐POSS (≈50 V). This low turn‐on voltage fulfills the requirements of safe operation for a majority of applications including human‐machine interfaces.^[^ ^8^ ^]^ These results thus demonstrate the effectiveness of the tri‐layer architecture comprising F‐POSS in promoting electroluminescence. We also compared the luminance of the F‐POSS‐enabled devices with other reported ones at the same electric fields (Figure 5f). It is seen that the luminance of the sandwiched composite with F‐POSS dramatically climbs to 672 cd m^−^^2^ as the applied electric field increases to 10 MV m^−1^, which is over 14 times higher than that of the commercial device.^[^ ^8^, ^68^ ^]^ Such an efficient luminance also significantly surpasses that using single‐layer P(VDF‐HFP)/F‐POSS composites (448 cd m^−2^), and outperforms other devices based on polymers including P(VDF‐HFP), polydimethylsiloxane (PDMS), and poly(vinylidene fluoride‐trifluoroethylene) [P(VDF‐TrFE)], which generally exhibit luminance below 300 cd m^−2^.^[^ ^4^, ^8^, ^9^, ^70^, ^71^, ^72^, ^73^, ^74^, ^75^, ^76^, ^77^, ^78^ ^]^ These comparisons further highlight the important role of F‐POSS in improving electroluminescence, demonstrating that the sandwiched polymer composites with F‐POSS hold great promise for optoelectronic applications.

## 3 Conclusion

In summary, we have achieved efficient energy storage and electroluminescence in low‐loss ferroelectric polymer composites comprising an organic–inorganic hybrid—perfluorinated polyhedral oligomeric silsesquioxane (F‐POSS). Both theoretical and experimental results reveal that F‐POSS exhibits intrinsic wide bandgap and strong carrier‐trapping properties, combined with favorable intermolecular interactions with the polymer that induce conformational changes. These benefits lead to significantly depressed energy loss and markedly enhanced electric breakdown strength without compromising the dielectric constant, giving rise to both high energy density and efficiency in the P(VDF‐HFP)/F‐POSS composites. The inclusion of F‐POSS into a tri‐layer device demonstrates further enhanced capacitive energy storage performance, achieving a giant energy density of over 32 J cm^−3^ with an ultrahigh efficiency of over 80%, significantly outperforming current ferroelectric polymers and being on par with the best composites reported ever. Moreover, such a design also boosts alternating current electroluminescence in the tri‐layer device, reaching over 670 cd m^−2^, which is over ten times higher than commercial products and superior to a variety of polymers. Coupled with good flexibility, ease of processing, and a vast choice of fluorinated organosilicons, these ferroelectric polymer composites show great promise for high‐energy‐density and optoelectronic applications, offering new possibilities for the design and realization of multifunctional electronics and energy devices.

## Conflict of Interest

The authors declare no conflict of interest.

## Supporting information



Supporting Information

## Data Availability

The data that support the findings of this study are available from the corresponding author upon reasonable request.
