# Supplementary material for: Perfluorinated Organosilicons Enabling Low‐Loss Ferroelectric Polymer Composites for Efficient Energy Storage and Electroluminescence
Source: Adv Sci (Weinh). 2025 Mar 6;12(17):2414380. doi: 10.1002/advs.202414380 (PMC12061332; doi:10.1002/advs.202414380)
Supplement: Supplementary file 1 — Supporting Information [file ADVS-12-2414380-s001.docx]

**Supporting information**

**Perfluorinated Organosilicons Enabling Low-loss Ferroelectric Polymer Composites for Efficient Energy Storage and Electroluminescence**

*Li Li, Zhubing Han, Hemant P Yennawar, Yunyun Cheng, Ting Han*, Rui Feng, Yang Zhang, Guanghui Zhao*, Qing Wang^*^ and Lijie Dong^*^*

Dr. L. Li, Dr. Y. Cheng, Dr. T. Han, Dr. R. Feng, Dr. Y. Zhang, Prof. L. Dong

Center for Smart Materials and Devices, State Key Laboratory of Advanced Technology for Materials Synthesis and Processing, Wuhan University of Technology, Wuhan, Hubei, 430070, P. R. China

Email: hanting9969@whut.edu.cn, dong@whut.edu.cn

Dr. L. Li, Dr. Z. Han, Dr. Y. Cheng, Prof. Q. Wang

Department of Materials Science and Engineering, The Pennsylvania State University, University Park, PA 16802, USA

Email: wang@matse.psu.edu

Dr. H. Yennawar

Department of Biochemistry and Molecular Biology, The Pennsylvania State University, University Park, PA 16802, USA

Prof. G. Zhao

Research Center for Materials Genome Engineering, International Materials Science and Engineering, Wuhan University of Technology, Wuhan, Hubei, 430070, P. R. China

Email: zhaogh@whut.edu.cn

**Keywords**: ferroelectric polymer, organosilicon, energy storage, electroluminescence

**Experimental methods**

**Synthesis of F-POSS**

0.01 mol *1H,1H,2H,2H-*perfluorooctyltriethoxysilane (POTS, Sigma-Aldrich, 99%) was added in a mixed solution of 540 mg distilled water, 3.0 mg NaOH and 20 mL ethanol, followed by vigorous stirring at 30 ℃ for 24 h. The white powders of compound were then obtained and washed with ethanol, then dried under vacuum at 60 °C for 24 hours. The yield was ~94%. The colorless crystals were grown by using a slow evaporation method from hexafluorobenzene solution of the compound at -5 ℃.^[1]^

**Fabrication of P(VDF-HFP)/F-POSS composite films**

0.5 g P(VDF-HFP) pellets (Sigma-Aldrich, 10 mol% of HFP) were dissolved into 10 mL DMAc and stirred for 30 h to obtain a homogeneous solution. A certain amount of F-POSS powders were added in with different concentrations. After sonication for 30 min, the mixture was heated to 60 ℃ with vigorous stirring for 24 h until the solution became clear. Following that, the mixed solution was cast onto a cleaned glass, then dried at 70 °C for 12 h and 120 °C under vacuum for 24 h. Thus, the P(VDF-HFP)/F-POSS films were obtained after being peeled off from the glass plates. Typical thickness of the films is 8~12 μm.

**Fabrication of tri-layered composite films for capacitors**

The fabrication of P(VDF-HFP)/QDs films was described elsewhere before.^[2]^ The tri-layered composite film was prepared by hot-pressing three layer of films following the order: from bottom to top: P(VDF-HFP)/QDs, P(VDF-HFP)/F-POSS and P(VDF-HFP)/QDs at 180 ℃ under 15 MPa for 20 min. For better characterizing the electric breakdown and field-dependent polarizations of the tri-layer films and considering the limit of voltage provided by our voltage amplifier, the thicknesses of the tri-layer films are controlled to be 14-16 μm, with each single layer 5-6 μm. Then, both sides of the films were sputtered with Au electrodes to fabricate the capacitors.

**Fabrication of tri-layered composites with phosphors for ACEL devices**

To prepare the tri-layered composites with phosphors, a layer-by-layer casting method is employed, instead of a hot-pressing procedure that may cause cracks of ITO glasses and failure of devices. To prepare the P(VDF-HFP)/F-POSS/phosphor single-layer films, 0.5 g P(VDF-HFP) pellets were firstly dissolved into 10 mL DMAc and stirred for 30 h to obtain a homogeneous solution. A certain amount of F-POSS powders were then added in to prepare P(VDF-HFP)/F-POSS mixed solution with 12 wt% F-POSS. After sonication for 30 min, the mixture was heated to 60 ℃ with vigorous stirring for 24 h until the solution became clear. Following that, certain amount of phosphor (ZnS: Cu) powders (which were filtrated in advance to remove large particles with diameter over 20 μm) were added in to prepare the three-component solution, keeping stirring to make the solution homogenous. The solution of P(VDF-HFP)/QDs composite (for the bottom layer) was firstly casted onto ITO-coated glass (cleaned by DI water and ethanol), then dried at 70 °C for 12 h and 120 °C under vacuum for 24 h. The P(VDF-HFP)/F-POSS/phosphor (for the middle layer) solution was then solution-casted onto it at room temperature, followed by drying at 70 °C for 12 h and 120 °C under vacuum for 24 h. The top layer of P(VDF-HFP)/QDs composite was then prepared similarly to the bottom layer. The typical thicknesses of the tri-layer films are 30-35 μm. Top side of the film was pasted with patterned copper tapes as electrodes to fabricate the EL device.

**Materials Characterization**

The single-crystal X-ray diffraction data was collected on a Rigaku Oxford Diffraction (ROD) Synergy Custom system comprising of MicroMax 007 rotating anode (CuKα) X-ray generator and HyPix-Arc 150 area detector. Computer programs used for the data collection and solving the crystal structure include CrysAlis PRO 1.171.41.108a (Rigaku OD, 2021), SHELXT (Sheldrick, 2015), SHELXL 2018/3 (Sheldrick, 2015), Olex2 1.3-ac4 (Dolomanov et al., 2009). Numerical absorption correction was based on Gaussian integration over a multifaceted crystal model, and empirical absorption correction was employed using spherical harmonics, implemented in SCALE3 ABSPACK scaling algorithm. CCDC#2100198 contains the supplementary crystallographic data for F-POSS can be obtained free of charge from The Cambridge Crystallographic Data Centre via [www.ccdc.cam.ac.uk/](http://www.ccdc.cam.ac.uk/) data_request/cif.

Chemical structures of F-POSS were revealed by an X-ray photoelectron spectroscopy (ESCALAB 250Xi, Thermo Fisher) and a Fourier transform infrared spectroscopy (Nicolet 6700) instruments. Matrix-assisted laser desorption/ionization time-of-flight (MALDI-TOF) mass spectrum was recorded on an ultraflextreme MALDI TOF/TOF Mass Spectrometer (Bruker Daltonics) in positive reflection mode. Nuclear magnetic resonance (NMR) data were collected using a Bruker AXS SMART APEX spectrometer, with chemical shifts reported in parts per million (δ ppm). ^1^H NMR ((CD3)2CO, 500 MHz): δ 1.00-1.25 (m, 16H), 2.15-2.44 (m, 16H); 19F NMR ((CD3)2CO, 470 MHz): δ -81.9 (24F), -116.7 (16F), -122.7 (16F), -123.7 (16F), -124.1 (16H), -127.1 (16F). Thermogravimetric analysis was performed by a STA449F3 instrument with a heating rate of 10 °C/min from 50 °C to 700 °C under a nitrogen atmosphere. Differential scanning calorimetry curves was recorded by a PYRIS1 instrument from Perkin Elmer with a heating rate of 10 °C/min from 50 °C to 200 °C under a nitrogen atmosphere. Field-emitted scaning electron microscopy (FESEM) images were taken using a JSM-5610LV (HITACHI, Japan) at 5 kV. The cross-section samples of composite films were prepared by cutting the films previously frozen by liquid nitrogen or by cutting with a knife. Transmission electron microscopy (TEM, Joel JEM-2001 F) images were obtained at an accelerating voltage of 200 kV.

**Devices Characterization**

For electrical measurements, both sides of the films were sputtered by gold as electrodes with a diameter of 3 mm and a thickness of 50 nm. F-POSS powders were hot-pressed into a disk prior to gold sputtering. A precise digital LCR meter (E4980A, Agilent) was used to record the frequency-dependent dielectric constant and loss spectra with a frequency range from 20 Hz to 2 MHz at room temperature. For high-field electric properties, a multiferroic ferroelectric test system (Premier II, Radiant) with a Sawyer-Tower circuit was utilized to measure the electric displacement–electric field (*D–E*) loops and leakage current density. Samples were subject to a triangular unipolar wave under 10 Hz at room temperature. A Keithley 6517A electrometer/high resistance meter and a TREK model 610C amplifier were used to measure electric resistivity.

For ACEL characterizations, alternating voltage signals (standard sine wave) with adjustable voltage and frequency were generated using a programmable AC power source (IT7622, ITECH). The luminance of the devices was measured by a luminance meter (TOPCON BM-7) with a fixed distance. EL spectra of the composite films were collected by a steady-state fluorescence system (Horiba PTI-QM800).

**Theoretical computations**

All the calculations were conducted based on the density-function theory (DFT) framework implemented in Gaussian 09W software.^[3]^ All geometries were optimized at a B3LYP/6-31G level and were also characterized as global minima by frequency analyses to achieve lowest-energy configurations before electronic properties calculation. All optimizations were done without any symmetry constraints. The frontier molecular orbital energies were calculated at a B3LYP/def2-TZVP level. The binding energies (*E*_bin_) between F-POSS and the three conformations were calculated by following equation: *E*_bin_= *E*_com_- *E*_F_ - *E*_p_, where *E*_com_, *E*_F_, *E*_p_ represent the energies of the P(VDF-HFP)/F-POSS complex, F-POSS and the polymer conformation chain, respectively. The molecular interactions were analyzed based on IGMH using Multiwfn 6.0.^[4,5]^ GaussView and VMD were used for visualizations.


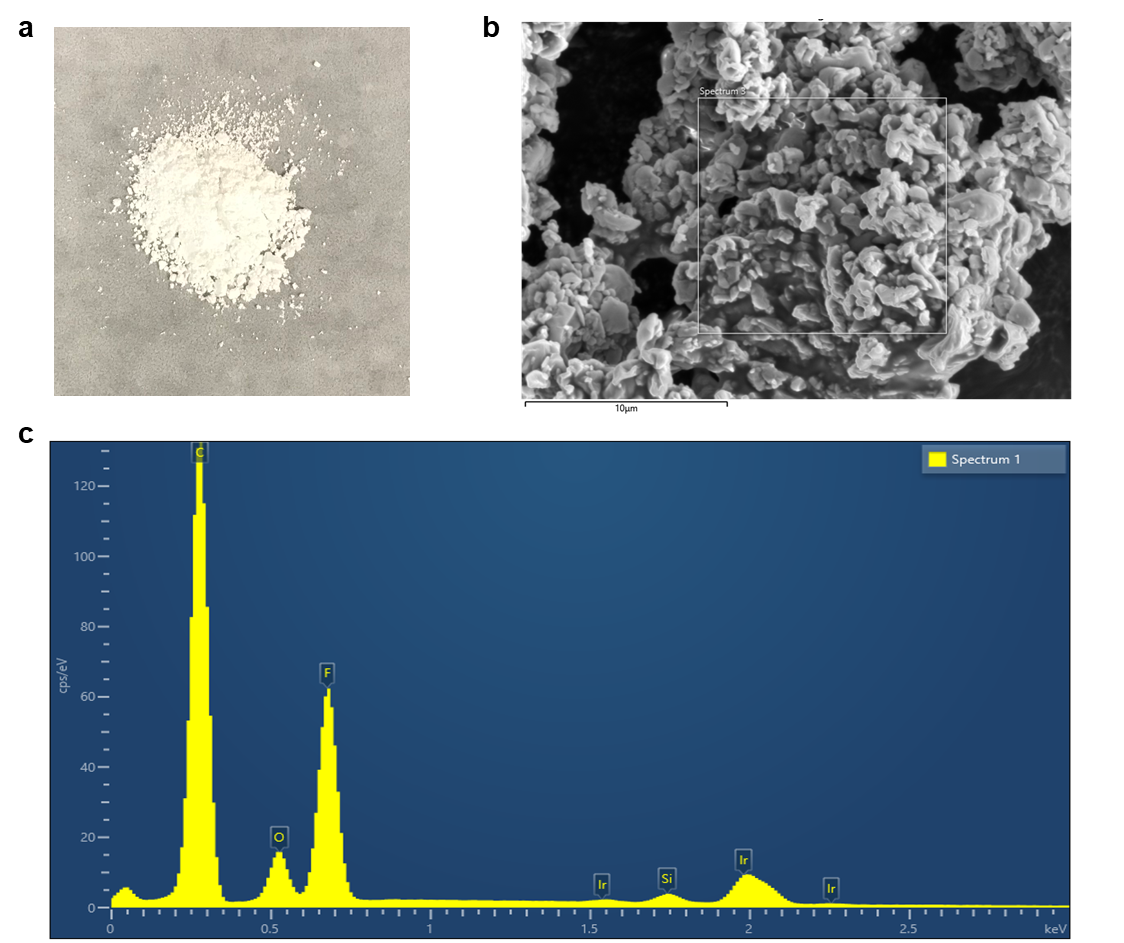


**Fig. S1** (a) The as-synthesized F-POSS powders. (b) SEM image of F-POSS powders. (c) EDS spectrum of F-POSS.

**Table S1** Summary of element compositions of F-POSS based on EDS results.

| Element | Line Type | Ratio | Wt% | Wt% Sigma | Atomic % |
| --- | --- | --- | --- | --- | --- |
| C | K series | 0.44041 | 59.49 | 0.08 | 69.59 |
| O | K series | 0.04749 | 7.27 | 0.05 | 6.39 |
| F | K series | 0.22164 | 30.92 | 0.07 | 22.86 |
| Si | K series | 0.02199 | 2.31 | 0.05 | 1.16 |
| Total: |  |  | 100.00 |  | 100.00 |


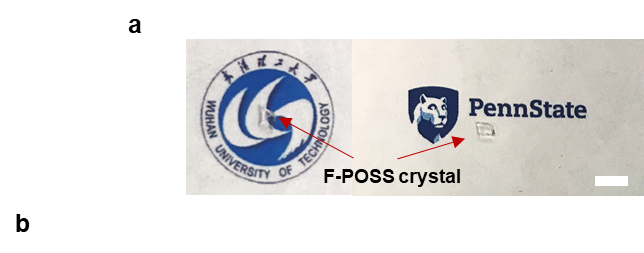


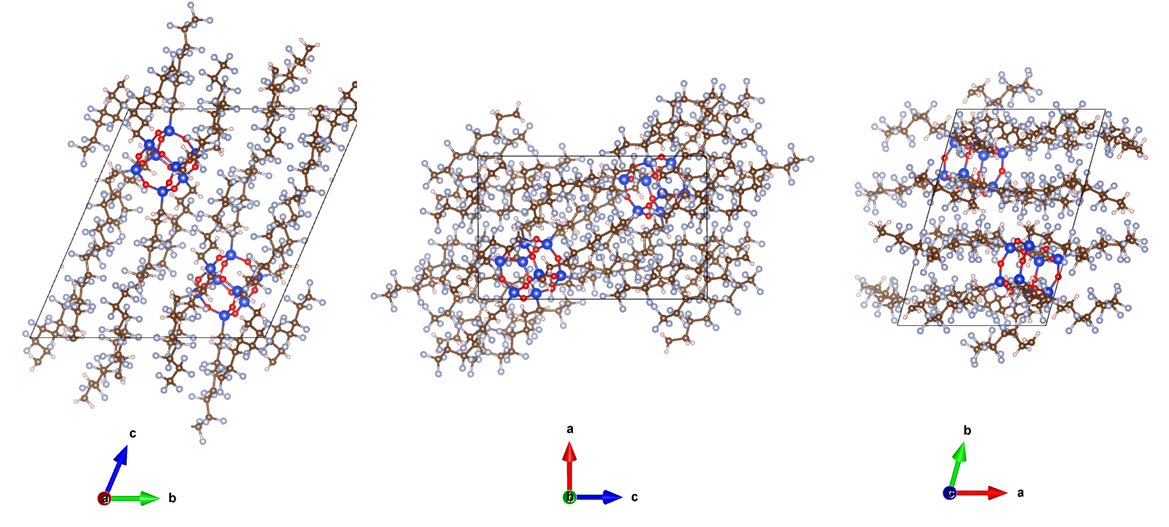


**Fig. S2** (a) Optical images of F-POSS crystals, scale bar 1 cm. (b) Schematic diagrams of F-POSS crystal structure at different view directions derived from single-crystal XRD data. The dark blue, dark red, light blue, brown and light red spheres represent silicon, oxygen, fluorine, carbon and hydrogen atoms, respectively.

**Table S2** Cell parameters of F-POSS crystal.

| Items | Results |
| --- | --- |
| Chemical formula | C64 H32 F104 O12 Si8 |
| Molecular weight | 3193.48 g/mol |
| Crystal system | Triclinic |
| Space group | P-1(2) |
| Lattice parameters | a=12.9406(3) Å， α=65.8550(15)°  b=21.2083(4) Å， β=83.4091(14)°  c=21.6825(3) Å， γ=73.2183(17)° |
| Cell ratios | a/b=0.6102， b/c=0.9781， c/a=1.6755 |
| Cell volume | 5198.82(17) Å^3^ |
| Density | 2.03992 g/cm^3^ |


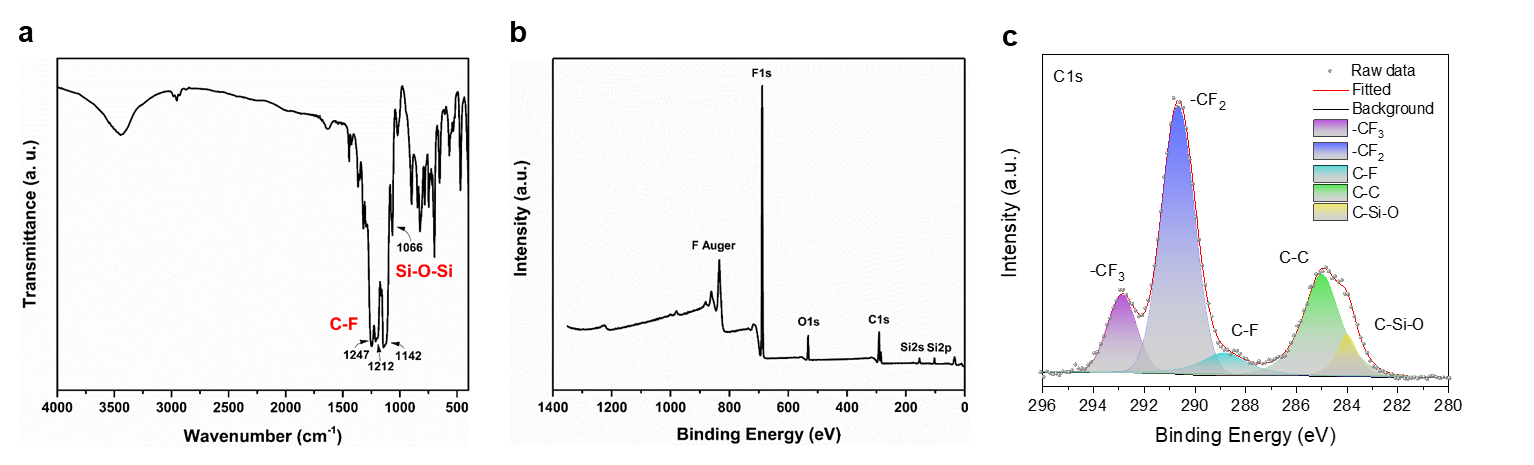


**Fig. S3** (a) FTIR spectrum of F-POSS. (b) XPS survey of F-POSS. (c) High-resolution XPS spectrum of C1s of F-POSS.


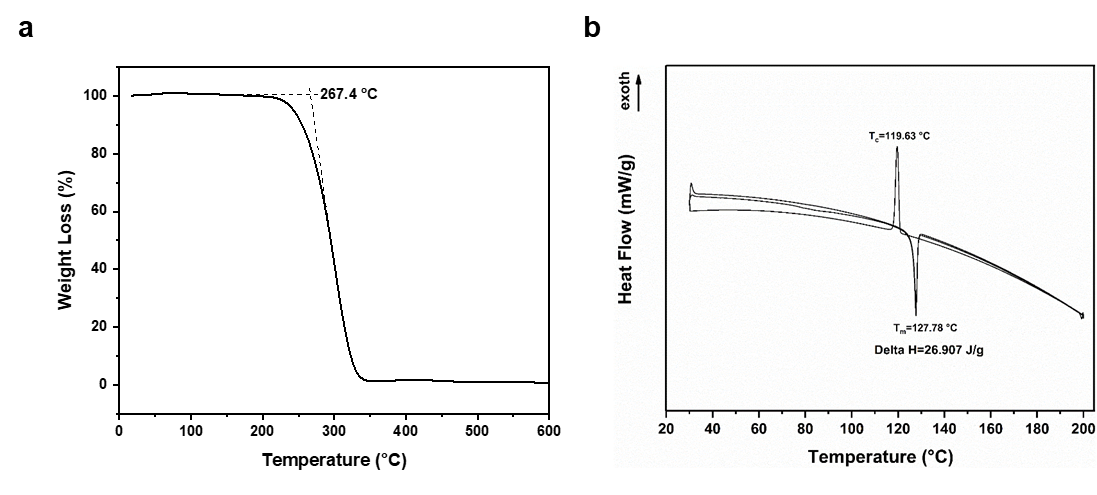


**Fig. S4** (a) Weight loss plot of F-POSS. (b) DSC curves of F-POSS.

We observe from TGA test that F-POSS just sublimes into atmosphere (N_2_) instead of decomposition at an onset temperature of ~267.4 °C (determined by drawing a tangent to the baseline before the decomposition and a tangent to the steepest part of the mass loss curve). There are no residues left after the heating procedure, also there is only one large weight loss in the whole process, which do not likely corresponds to the decomposition of conventional POSS or fluorinated groups-contained compounds (that generally show several stages of loss with residues). Similar phenomena have been previously reported in other fluorinated organosilicons.^[1]^


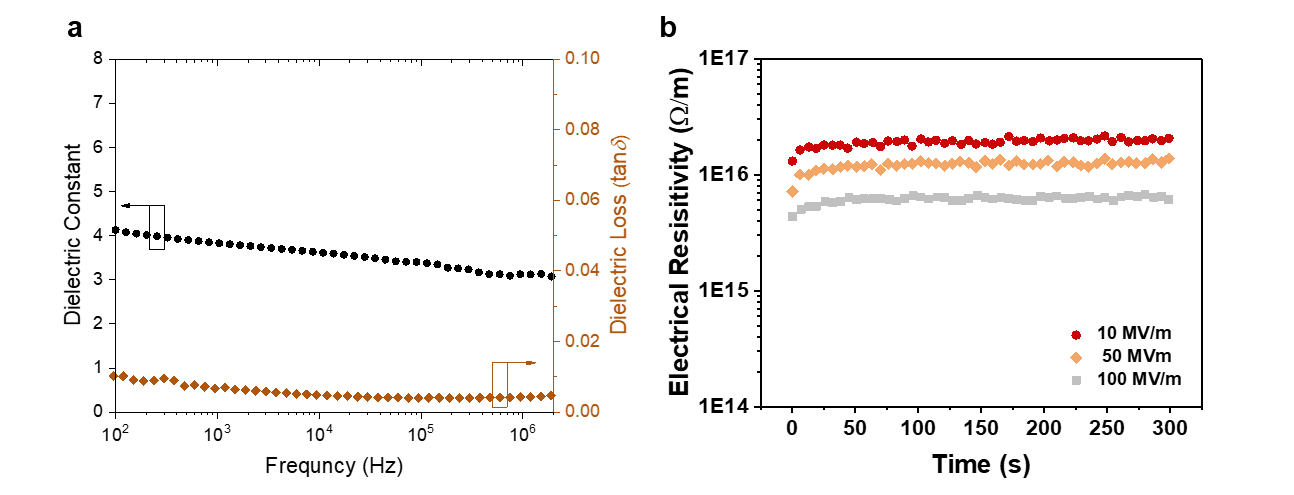


**Fig. S5** (a) Frequency-dependent dielectric constant and loss spectra of F-POSS. (b) Time-dependent electric resistivity of F-POSS at varied electric fields.


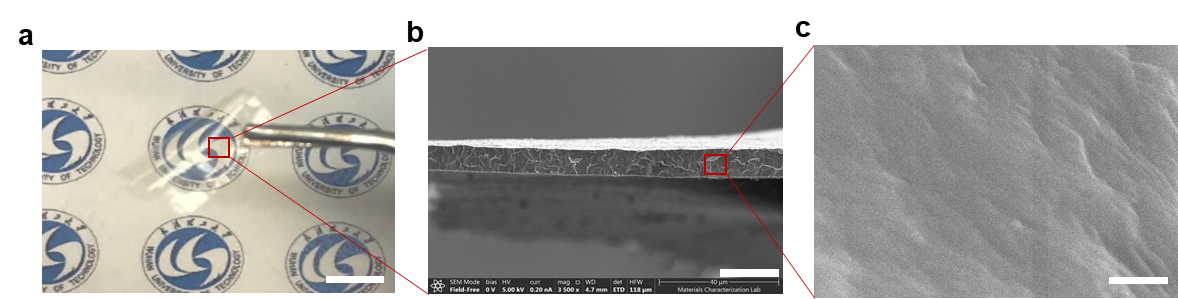


**Fig. S6** (a) Optical image of the P(VDF-HFP)/F-POSS composite film. Scale bar, 1 cm. (b) Cross-section SEM image of the film. Scale bar, 20 μm. (c) Enlarged view of (b). Scale bar, 100 nm.


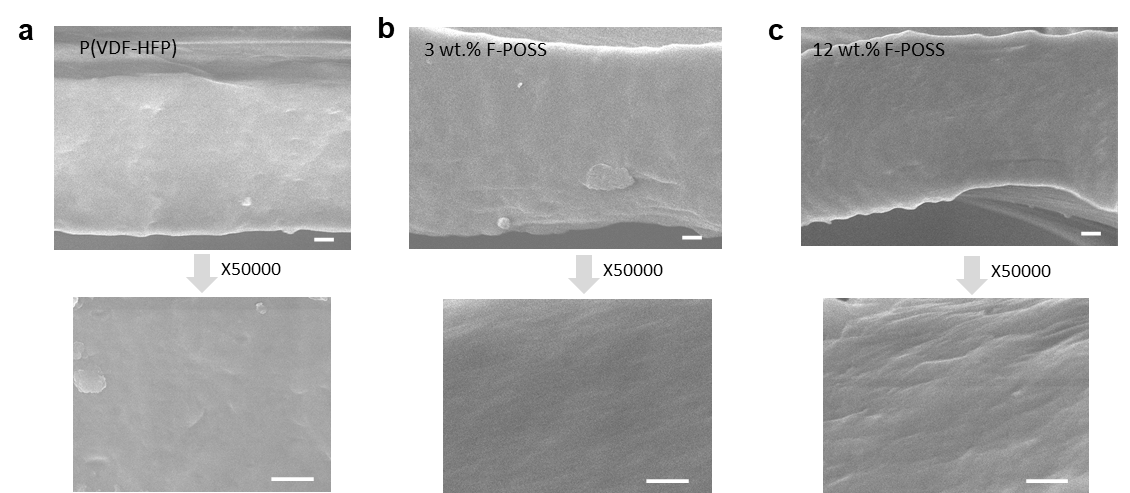


**Fig. S7** Cross-section SEM images of (a) pristine P(VDF-HFP), (b) P(VDF-HFP)/3 wt% F-POSS and (c) P(VDF-HFP)/12 wt% F-POSS. Scale bars, 1 μm. Below are corresponding enlarged view of these images. Scale bars, 100 nm.


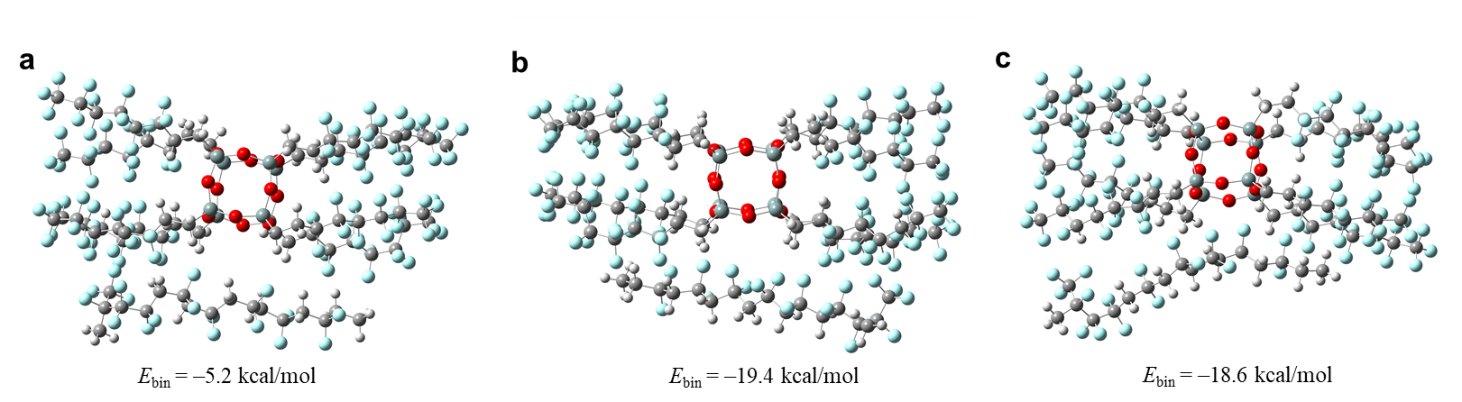


**Fig. S8** Optimized molecular configurations of P(VDF-HFP)/F-POSS complexes with varied conformations of P(VDF-HFP): (a) *tgtg’*, (b) *tttt* and (c) *tttgtttg’*. The balls colored by white, grey, light grey represent H, C, O, F and Si atoms, respectively.


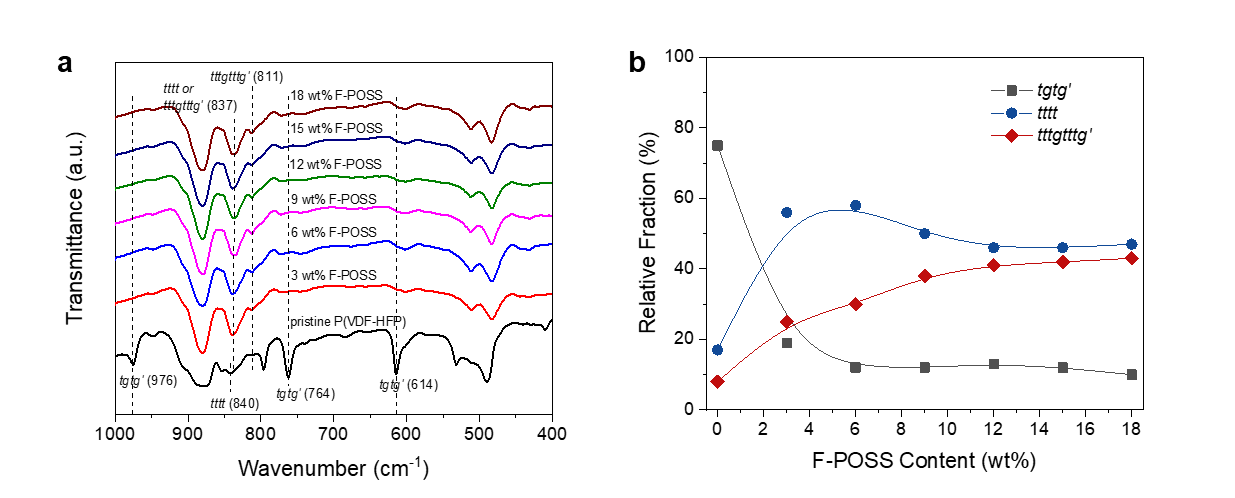


**Fig. S9** (a) FTIR spectra of pristine P(VDF-HFP) and P(VDF-HFP)/F-POSS composites with various F-POSS contents. (b) Relative fraction of the three conformations (*tgtg’*, *tttt* and *tttgtttg’*) of P(VDF-HFP) as a function of F-POSS content.


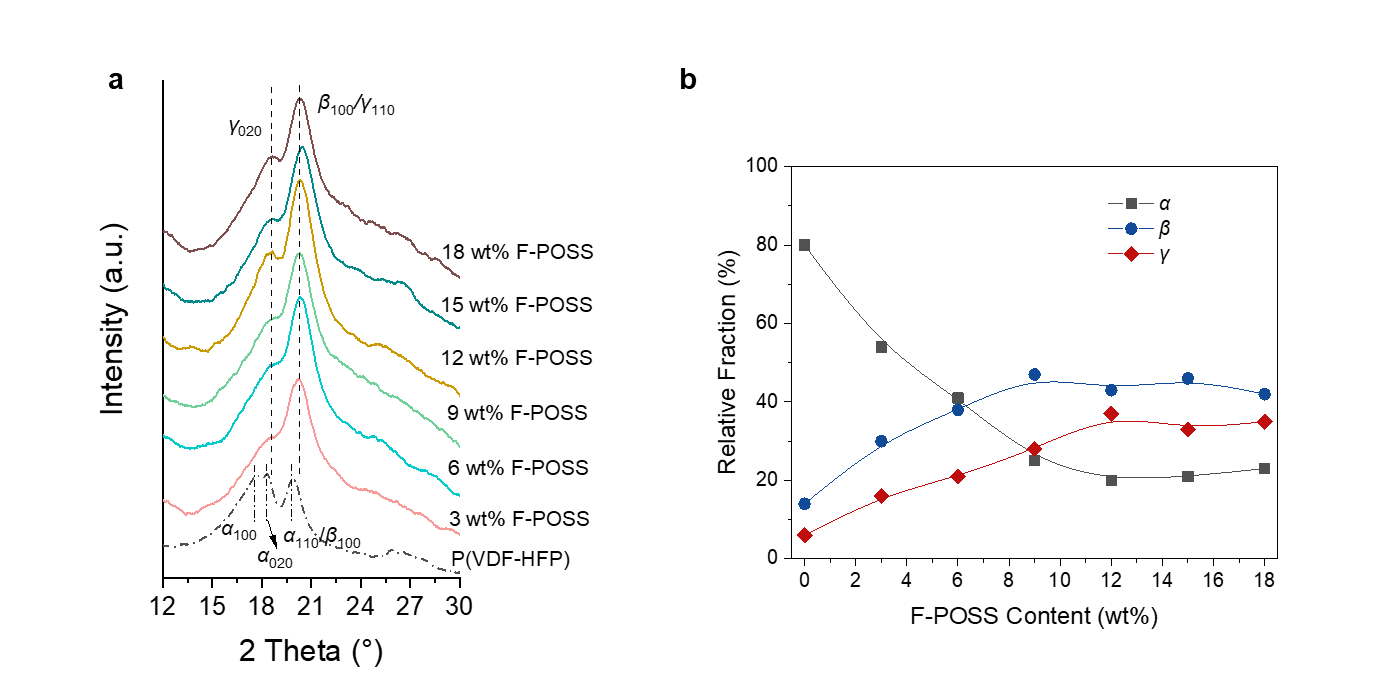


**Fig. S10** (a) XRD patterns of pristine P(VDF-HFP) and P(VDF-HFP)/F-POSS composites with various F-POSS contents. (b) Relative fraction of the three phases (*α*, *β*, *γ*) of P(VDF-HFP) as a function of F-POSS content.


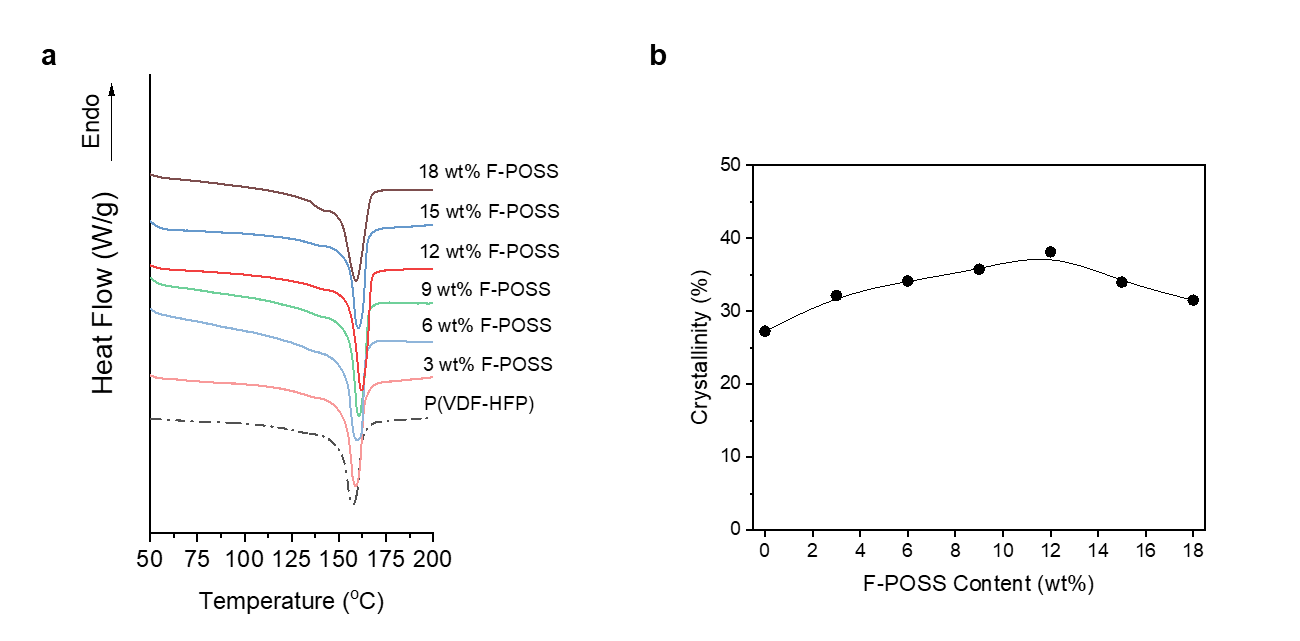


**Fig. S11** (a) DSC curves of pristine P(VDF-HFP) and P(VDF-HFP)/F-POSS composites with various F-POSS contents. (b) Crystallinity of P(VDF-HFP) as a function of F-POSS content derived from (a).


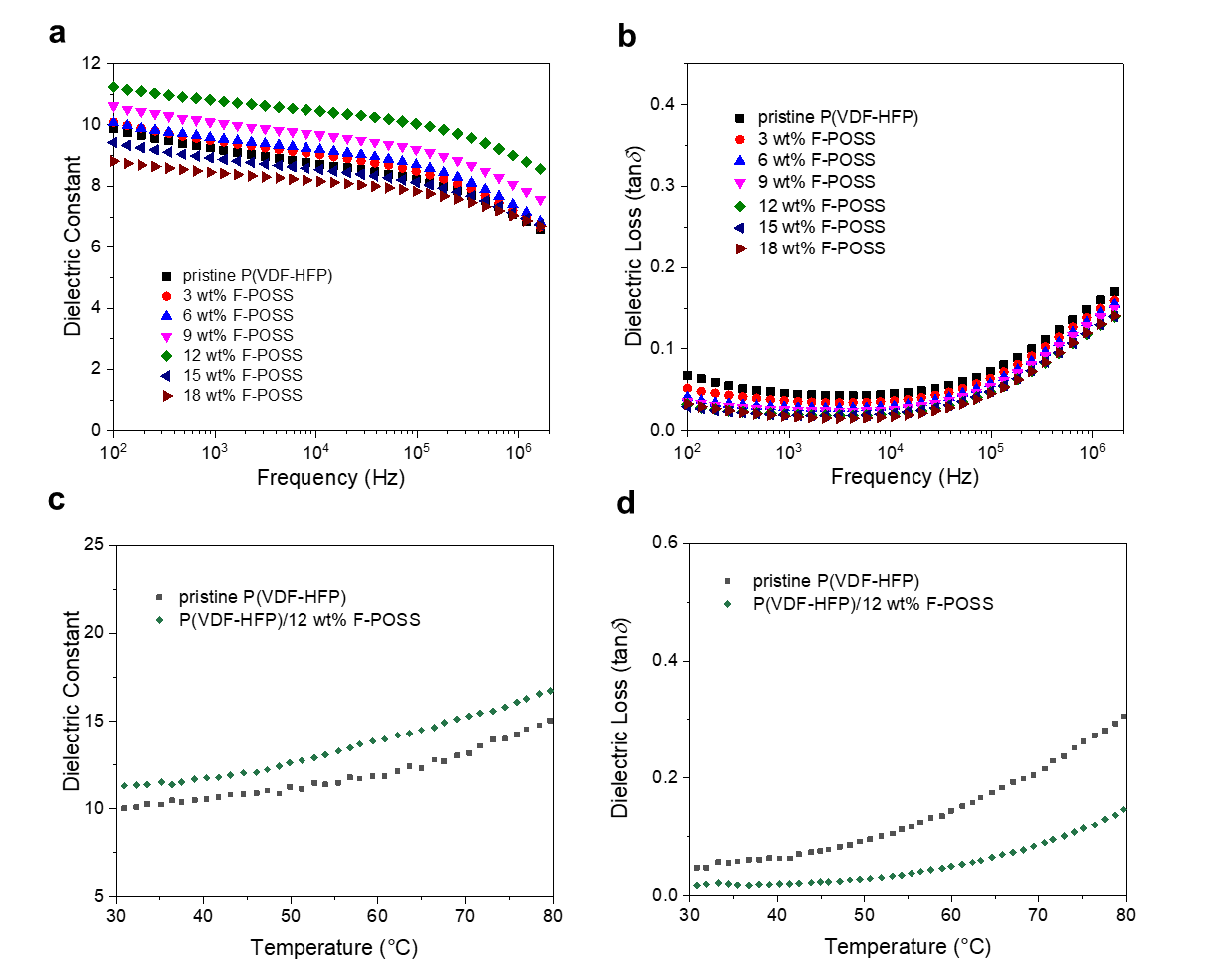


**Fig. S12** Frequency-dependent (a) dielectric constant and (b) loss spectra of pristine P(VDF-HFP) and the composites with various F-POSS contents, at room temperature. Temperature-dependent (c) dielectric constant and (d) loss spectra of pristine P(VDF-HFP) and P(VDF-HFP)/ 12 wt% F-POSS, at 1 kHz. It is seen that both K and tan*δ* increase upon increasing temperatures in the (PVDF-HFP) composite with 12 wt% F-POSS, due to the ferroelectric nature of the polymer matrix. Comparatively, the composite exhibits lower tan*δ* than the pristine polymer over the entire temperature range, which also reflects the role of F-POSS in reducing conduction losses in the ferroelectric polymer.


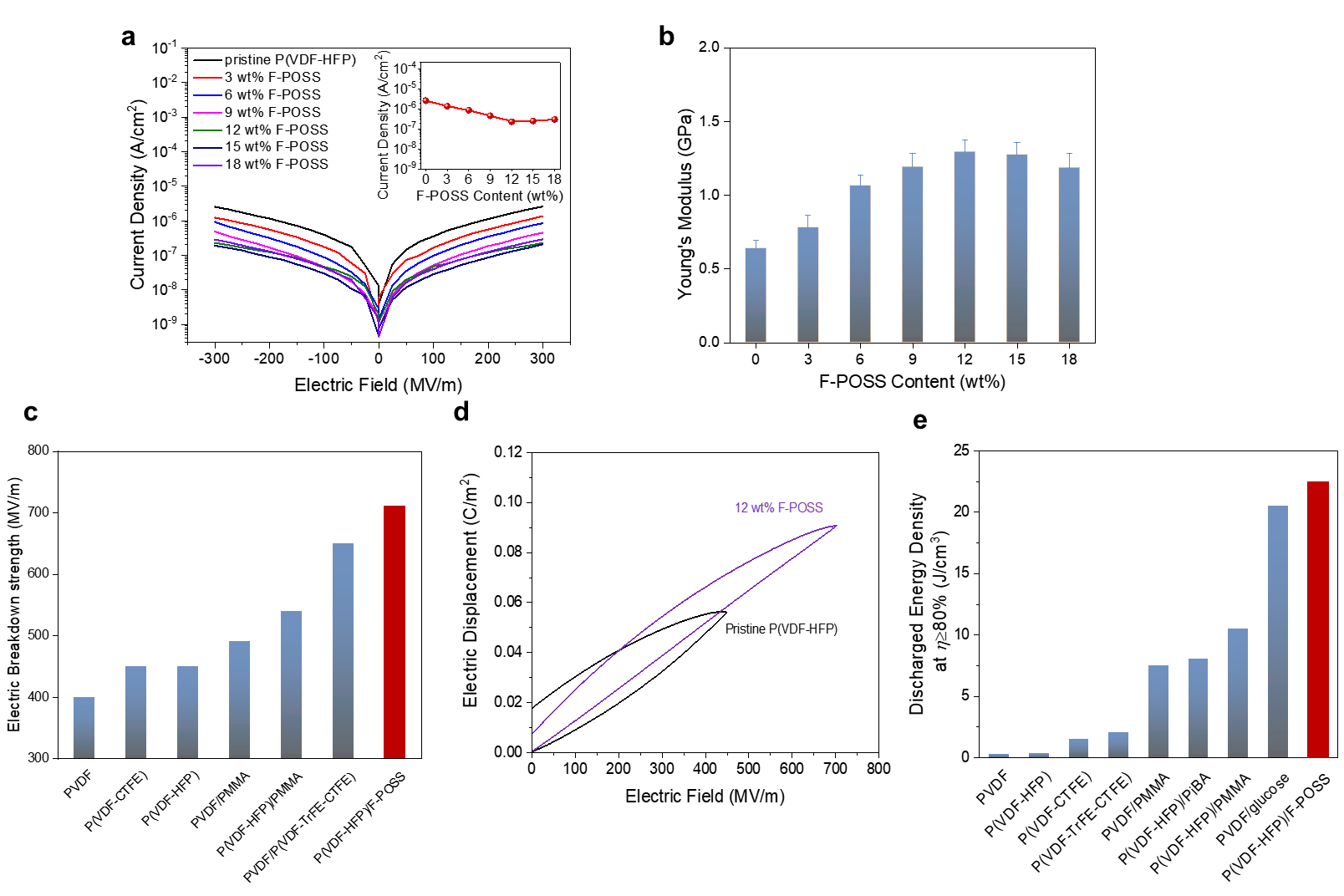


**Fig. S13** (a) Electric-field dependent current density and (b) Young’s modulus of P(VDF-HFP) composites as a function of F-POSS content. (c) Comparison of electric breakdown strength between this work and other reported results. (d) Electric displacement-electric field loops of pristine P(VDF-HFP) and P(VDF-HFP)/12 wt% F-POSS composite. (e) Comparison of discharged energy density at ≥80% efficiency between this work and other reported results.^[6–13]^


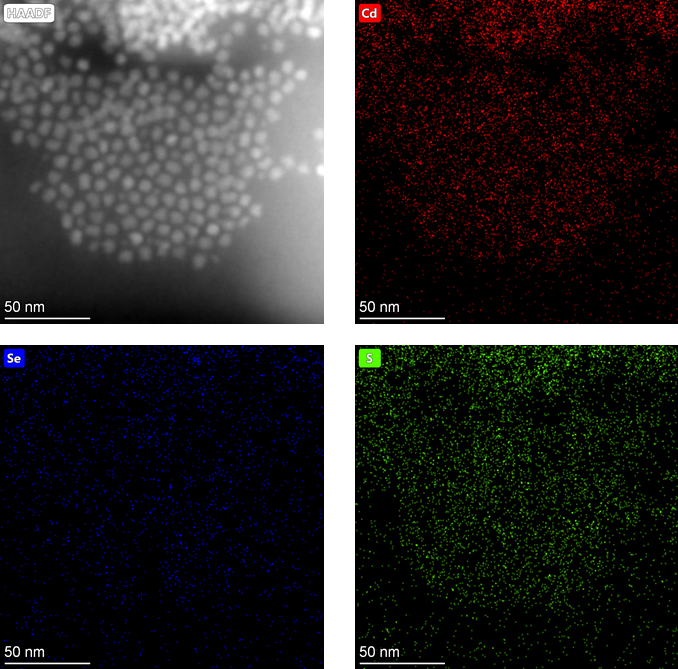


**Fig. S14** TEM image and EDS mappings (Cd, Se, S) of the Cd_1-x_Zn_x_Se_1-y_S_y_ QDs. Scale bars, 50 nm.


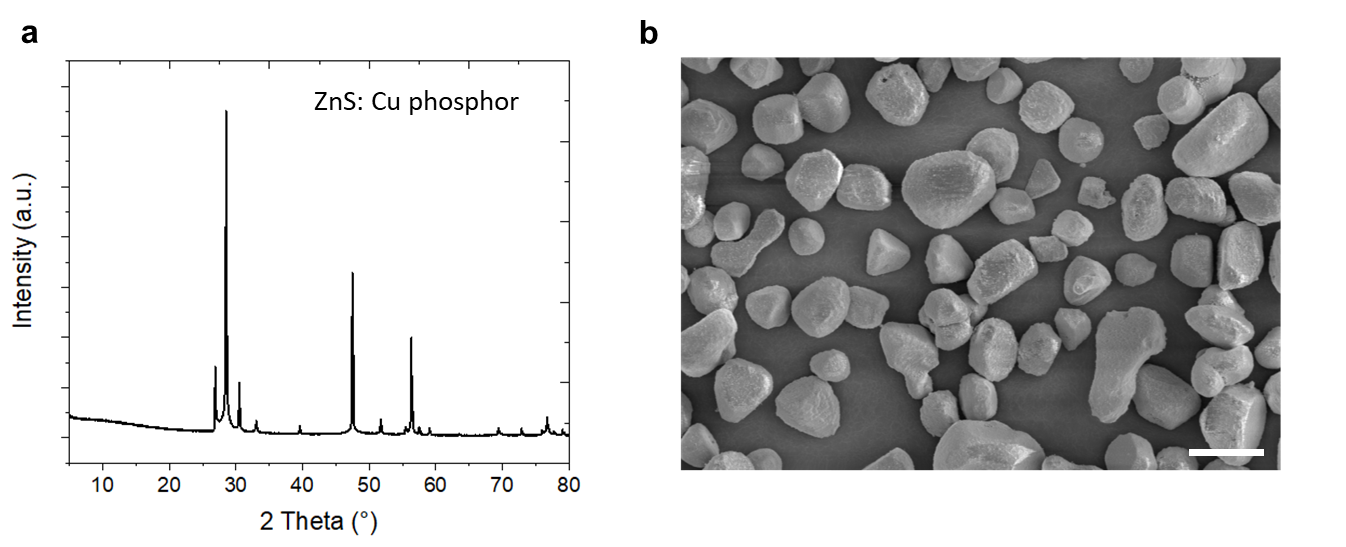


**Fig. S15** (a) XRD pattern of the as-received commercial phosphors (ZnS: Cu). (b) SEM image of the phosphor particles. Scale bar, 50 μm.

There is a dielectric mismatch between the top/bottom and middle layers, resulting in an uneven distribution of the electric field in the vertical direction, as shown by the following formula:

$E_{\mathrm{out}}=\frac{V}{2d_{1}+\frac{\varepsilon_{1}}{\varepsilon_{2}}d_{2}} , E_{\mathrm{int}}=\frac{V}{2d_{1}+\frac{\varepsilon_{2}}{\varepsilon1}d_{2}}$ (S1)

Where $V$ is the applied external voltage, $E_{out}$and $E_{int}$are the electric field strengths of the top/bottom layer and the middle layer, respectively, *ε*₁ and *ε*₂ are the dielectric constants of the top/bottom layer and the middle layer, respectively, and *d*₁ and *d*₂ are the thicknesses of the top/bottom layer and the middle layer, respectively. By controlling the thicknesses of the three layers to be consistent, i.e., $d_{1}$ = $d_{2}$, formula (1) can be simplified as:

$E_{out}=\frac{{3E}_{tri}}{2+\frac{\varepsilon_{1}}{\varepsilon_{2}}} , E_{int}=\frac{{3E}_{tri}}{2+\frac{\varepsilon_{2}}{\varepsilon1}}$ (S2)

Where $E_{tri}$is the overall breakdown strength of the tri-layer film.

Since $\varepsilon_{1}> \varepsilon_{2}$[the top/bottom layer, i.e., P(VDF-HFP)/QDs, has a higher *K* than the middle layer, i.e., P(VDF-HFP)/F-POSS], $E_{int}$ will be higher than $E_{out}.$ Furthermore, the higher *K* of P(VDF-HFP)/F-POSS than the phosphors will further concentrate the electric field on phosphor particles, therefore enabling higher EL intensity at low voltages.


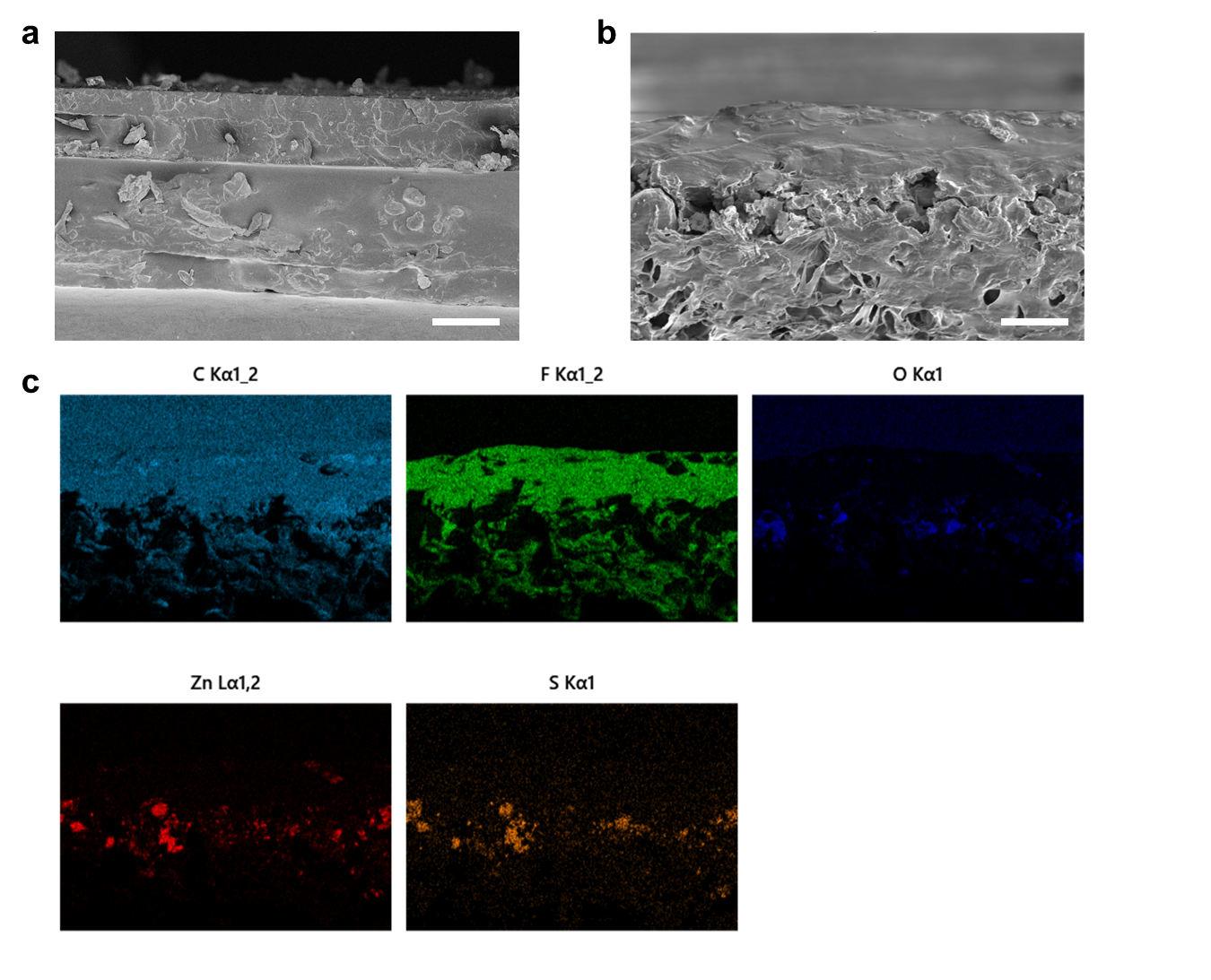


**Fig. S16** Cross-section images of the tri-layered composite films with phosphors (40 wt%), with (a) samples frozen by liquid nitrogen and (b) samples cut with knife. Scale bars, 10 μm. (c) EDS mapping of C, F, O, Zn and S based on (b). The films generally show good morphological integrality, with fractures caused by liquid-nitrogen freezing. In this case, the phosphor particles were well encapsulated in the polymer and make it difficult to acquire high-quality EDS mapping for them. To better probe the element distributions, the cross-sectioned samples were prepared by simply cutting to protrude the particles in the middle layer.

**Fig. S17** Normalized EL intensity of the composite films with P(VDF-HFP) and P(VDF-HFP)/F-POSS as a function of phosphor content.


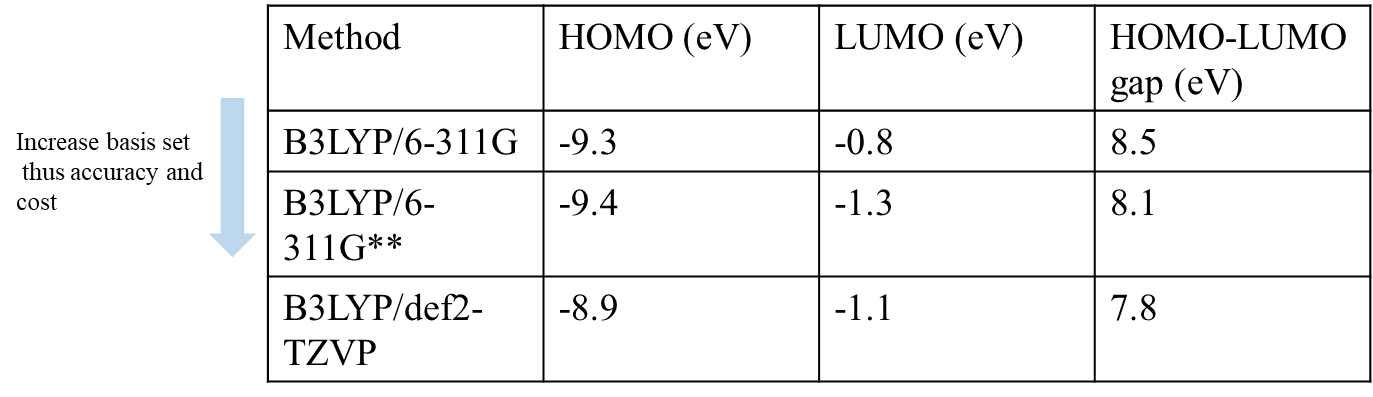


**Table S3**. DFT calculations of HOMO, LUMO and HOMO-LUMO gap of P(VDF-HFP) using different computing methods.

To achieve higher accuracy of computations, we study different basis sets from 6-311G to larger levels such as 6-311G** and def2-TZVP, to calculate the HOMO, LUMO levels and gap of P(VDF-HFP). It is seen that the HOMO-LUMO gap decreases with increasing the basis set, from 8.5 eV to 7.8 eV. The difference is not large, suggesting that the basis sets are generally good under such functional within the density-functional theory framework.

We note that there is a large difference between the theoretical gap and real one (*e.g.*, 5.5~6.5 eV) for P(VDF-HFP) mainly determined from UV-vis absorption. The reasons lie in at least three aspects: (1) Excitonic effects. The calculated gap for P(VDF-HFP) is fundamental gap (*E*_fund_), while the experimentally derived one is optical gap (*E*_opt_). Note that *E*_fund_ is always larger than the *E*_opt_, since *E*_fund_= *E*_opt_+*E*_b_, where *E*_b_ is the binding energy of electron-hole pair.^[14]^ (2) In practical, there are electronic defect states brought by structural defects or impurities that are unavoidable in the real polymers, which further decrease the optical gap. (3) The theoretical computation limitations. Currently developed calculation methods are generally not well fitted to the polymer or molecular systems, compared to the inorganic crystalline materials, due to effects like not fully involving electron correlations, neglecting inter-molecular interactions, *etc*. This can lead to overestimated bandgap calculation results. Since we are considering the intrinsic electronic structures of P(VDF-HFP) and F-POSS, it is appropriate to just focus on the theoretical side of fundamental gaps to compare them at a same level, to provide a theoretical support of correlating the electronic behaviors with the electrical conductions.

References

[1] J. M. Mabry, A. Vij, S. T. Iacono, B. D. Viers, *Angew. Chem. Int. Ed.* **2008**, *47*, 4137.

[2] L. Li, J. Cheng, Y. Cheng, T. Han, Y. Liu, Y. Zhou, G. Zhao, Y. Zhao, C. Xiong, L. Dong, Q. Wang, *Adv. Mater.* **2021**, *33*, 2102392.

[3] M. Frisch, G. W. Trucks, H. B. Schlegel, G. E. Scuseria, M. A. Robb, J. R. Cheeseman, G. Scalmani, V. Barone, B. Mennucci, Ga. Petersson, *Gaussian 09, Revision D. 01*, Gaussian, Inc., Wallingford CT, **2009**.

[4] T. Lu, Q. Chen, *J. Comput. Chem.* **2022**, *43*, 539.

[5] T. Lu, F. Chen, *J. Comput. Chem.* **2012**, *33*, 580.

[6] Y. Zhang, L. Li, X. Li, R. Feng, T. Zhao, M. Pan, L. Dong, *Adv. Funct. Mater.* **2023**, *33*, 2300555.

[7] Prateek, V. K. Thakur, R. K. Gupta, *Chem. Rev.* **2016**, *116*, 4260.

[8] W. Sun, X. Lu, J. Jiang, X. Zhang, P. Hu, M. Li, Y. Lin, C.-W. Nan, Y. Shen, *J. Appl. Phys.* **2017**, *121*, 244101.

[9] B. Luo, X. Wang, H. Wang, Z. Cai, L. Li, *Compos. Sci. Technol.* **2017**, *151*, 94.

[10] R. Wang, H. Xu, S. Cheng, J. Liang, B. Gou, J. Zhou, J. Fu, C. Xie, J. He, Q. Li, *Energy Storage Materials* **2022**, *49*, 339.

[11] T. Wang, X. Shi, R. Peng, G. Dong, H. Liu, B. Chen, M. Guan, Y. Zhao, B. Peng, C. Zhou, S. Yang, W. Qu, Y. Zhang, Z. Zhou, X. Ding, H. Wu, H. Huang, M. Liu, *Nano Energy* **2023**, 108511.

[12] H. Li, Z. Xie, C. Yang, J. Kwon, A. Lainé, C. Dun, A. V. Galoustian, X. Li, P. Liu, J. J. Urban, Z. Peng, M. Salmeron, R. O. Ritchie, T. Xu, Y. Liu, *Nano Energy* **2023**, *113*, 108544.

[13] K. Liu, Y. Liu, W. Ma, N. Takesue, C. Samart, H. Tan, S. Jiang, Z. Dou, Y. Hu, S. Zhang, H. Zhang, *Chem. Eng. J.* **2022**, *446*, 136980.

[14] J.-L. Bredas, *Mater. Horiz.* **2013**, *1*, 17.
